# Pseudo‐obstruction, stroke, and mitochondrial dysfunction: A lethal combination

**DOI:** 10.1002/ana.24736

**Published:** 2016-09-19

**Authors:** Yi Shiau Ng, Catherine Feeney, Andrew M. Schaefer, Carol Ellen Holmes, Paula Hynd, Charlotte L. Alston, John P. Grady, Mark Roberts, Mellisa Maguire, Alexandra Bright, Robert W. Taylor, Yan Yiannakou, Robert McFarland, Doug M. Turnbull, Gráinne S. Gorman

**Affiliations:** ^1^ Wellcome Trust Centre for Mitochondrial Research, Institute of Neuroscience, Newcastle UniversityNewcastle upon TyneUnited Kingdom; ^2^ Department of RadiologyThe Newcastle upon Tyne Hospitals NHS Foundation Trust, Royal Victoria InfirmaryNewcastle upon TyneUnited Kingdom; ^3^ The Greater Manchester Neuroscience Centre, Salford Royal NHS Foundation TrustSalfordUnited Kingdom; ^4^ Department of NeurologyThe Leeds Teaching Hospitals NHS TrustWest YorkshireUnited Kingdom; ^5^ Department of GastroenterologyCounty Durham and Darlington NHS Foundation TrustDurhamUnited Kingdom

## Abstract

**Objectives:**

The m.3243A>G *MTTL1* mutation is the most common cause of mitochondrial disease; yet there is limited awareness of intestinal pseudo‐obstruction (IPO) in this disorder. We aimed to determine the prevalence, severity, and clinical outcome of patients with m.3243A>G‐related mitochondrial disease manifesting with IPO.

**Methods:**

In this large, observational cohort study, we assessed the clinical, molecular, and radiological characteristics of patients with genetically determined m.3243A>G‐related mitochondrial disease, who presented with severe symptoms suggestive of bowel obstruction in the absence of an occluding lesion.

**Results:**

Between January 2009 and June 2015, 226 patients harbouring the m.3243A>G mutation were recruited to the Medical Research Council Centre Mitochondrial Disease Patient Cohort, Newcastle. Thirty patients (13%) presented acutely with IPO. Thirteen of these patients had a preceding history of stroke‐like episodes, whereas 1 presented 27 years previously with their first stroke‐like episode. Eight patients developed IPO concomitantly during an acute stroke‐like episode. Regression analysis suggested stroke was the strongest predictor for development of IPO, in addition to cardiomyopathy, low body mass index and high urinary mutation load. Poor clinical outcome was observed in 6 patients who underwent surgical procedures.

**Interpretation:**

Our findings suggest, in this common mitochondrial disease, that IPO is an under‐recognized, often misdiagnosed clinical entity. Poor clinical outcome associated with stroke and acute surgical intervention highlights the importance of the neurologist having a high index of suspicion, particularly in the acute setting, to instigate timely coordination of appropriate care and management with other specialists. Ann Neurol 2016;80:686–692

Mitochondrial diseases are an important group of inherited neurometabolic disorders characterized by marked genotypic and phenotypic heterogeneity.^1^ Once considered rare, it is now recognized that the prevalence of mitochondrial DNA (mtDNA) disease is sizeable at 1 in 5,000 of the population.^2^

The most common form of mitochondrial disease is attributed to the pathogenic mtDNA point mutation m.3243A>G in the mt‐tRNA leucine gene (*MTTL‐1*), estimated to be present in approximately 1 in 400 of the population.^3^, ^4^ Clinical syndromes classically associated with the phenotypic expression of the m.3243A>G mutation include mitochondrial encephalomyopathy, lactic acidosis, and stroke‐like episodes (MELAS), maternally inherited deafness and diabetes (MIDD), and chronic progressive external ophthalmoplegia. However, the majority of patients do not manifest as discrete syndromes, but, moreover, a wide overlapping spectrum of clinical features that invariably exhibit multiorgan involvement.^5^

Symptoms arising from gastrointestinal dysmotility in patients with mitochondrial disease are increasingly recognized and often include dysphagia, abdominal pain, abdominal distention, and constipation.^6^, ^7^, ^8^ Although gastrointestinal involvement, manifesting as intestinal pseudo‐obstruction (IPO), has been reported previously 9, 10, 11, 12, 13 and indeed is a recognized feature of the rare neurogastrointestinal encephalopathy (MNGIE) syndrome,^14^, ^15^ there remains a limited awareness of the severity of IPO in m.3243A>G‐related mitochondrial disease.^16^, ^17^

We detail the clinical, molecular, and radiological characteristics of patients harboring the m.3243A>G mutation manifesting with different clinical phenotypes, who presented with marked impairment in intestinal motility. We sought to determine the prevalence, severity, and outcomes of IPO in patients known to harbor the m.3243A>G mutation and devise a simple management algorithm that outlines the acute assessment and treatment of these patients.

## Subjects and Methods

### Study Design and Participants

Patients with mitochondrial disease attributed to the m.3243A>G mutation in *MTTL1* were enrolled to the UK Medical Research Council (MRC) Centre Mitochondrial Disease Patient Cohort (REC reference number: 13/NE/0326, approved by the NRES Committee North East–Newcastle and North Tyneside 2) in Newcastle, between April 1, 2009 and June 30, 2015. The Newcastle Mitochondrial Disease Adult Scale (NMDAS), a validated disease‐specific rating scale,^18^ was used to screen for gastrointestinal symptoms in patients harboring the m.3243A>G mutation in addition to interrogation of their case notes during hospital admission. IPO was defined based upon clinical symptoms (protracted nausea and vomiting, abdominal pain, and marked abdominal distension in association with severe constipation) and radiological findings (evidence of dilated bowel loops; small and/or large bowel, with or without gastric distension).^19^ This study was approved and performed under the ethical guidelines issued by our institution and complied with the Declaration of Helsinki.

### Molecular Genetic Studies

Total DNA was extracted from muscle, urine, and blood by standard procedures. Pyrosequencing (PSQ) was used to quantify the m.3243G>A heteroplasmy levels with mutation‐specific PSQ primers according to Genbank Accession number NC_012920.1: 5′biotinylated forward: m. 3143‐3163; reverse: m.3331‐3353; and reverse pyrosequencing primer: m.3244‐3258 (IDT, Coralville, USA).

### Outcomes

Interrogation of the clinical, molecular, and radiological (abdominal radiograph; computed tomography abdomen and pelvis) investigations of adult patients with m.3243A>G‐related mitochondrial disease manifesting with severe IPO was performed. Cause of death over the last 6 years (April 1, 2009–June 30, 2015 inclusive) was reviewed in all patients presenting with IPO and identified as harboring the m.3243A>G mtDNA point mutation.

### Statistical Analysis

Data were presented as mean ± standard deviation for continuous data that were normally distributed and as median for nonparametric, continuous data (mtDNA heteroplasmy). Ninety‐five percent confidence limits were calculated and described elsewhere.^20^ Cox regression analysis was used to identify putative factors that may predict development of IPO, and hazard ratios (HRs) were calculated. Statistical significance was determined at *p* < 0.05. Data were managed and analysed with IBM SPSS for Windows (version 22; IBM Corp, Armonk, NY).

## Results

### Participant Characteristics

Between April 1, 2009 and June 30, 2015, 1,239 patients were enrolled to the UK Medical Research Council (MRC) Centre Mitochondrial Disease Patient Cohort. Two hundred and twenty‐six patients (215 adult patients) with genetically determined m.3243A>G‐related mitochondrial disease were under active surveillance in Newcastle, with 14 patients living within North East England. Thirty patients (27 pedigrees, 13 men; mean age: 42.5 ± 16.3 years [range, 10–74]; mean body mass index [BMI]: 20.5 ± 4.3 [range, 10.6–27.8]; mean NMDAS score: 46 ± 17 [range, 16–82]) were identified with severe IPO (Supplementary Table). The mean age of establishing a genetic diagnosis of mitochondrial disease and IPO was 35.5 ± 15.6 (range, 6.9–62.8) and 38.4 ± 16.2 (range, 9.3–65.7) years, respectively. There was no statistical difference in mean age, BMI, age of establishing a diagnosis of mitochondrial disease, and IPO between sexes. Median urine and blood m.3243A>G mutation heteroplasmy levels were 81% (range, 35–99) and 26% (range, 5–61), respectively. Fourteen patients (47%) manifested with MELAS syndrome, 8 of whom developed IPO concomitantly during an acute stroke‐like episode. Sixteen patients had diabetes mellitus (53%), 9 of whom manifested with a MIDD syndromic phenotype. Notably, 1 patient developed recurrent IPO preceding their first stroke‐like episode by over two decades (subject 30; Supplementary Table). Twenty patients (67%) had at least a resting blood lactate level above the normal range (median, 3.7 mmol/l; range, 1.7–14.0).

### Radiological Features of IPO

**Figure 1:**
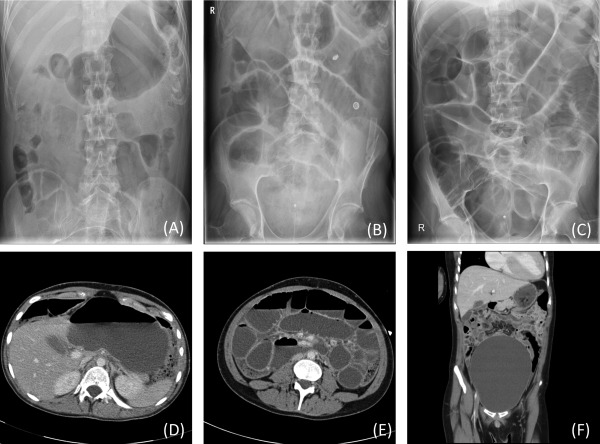
Radiological imaging studies. Abdominal radiograph (A–C) showed an evolution of intestinal pseudo‐obstruction involving both small and large bowels (radiograph performed on admission, day 5, and 10, respectively); computed tomography abdomen and pelvis (D) and (E) showed dilated, fluid‐filled stomach and small bowel loops; (F) very distended bladder and fecalization of small bowel.

Radiological investigations for 26 patients with acute presentation of IPO were available for analysis. Findings consistent with gastrointestinal dysmotility involving the whole gastrointestinal tract were observed and manifested as dilatation of the stomach and small and large bowels concomitantly (n = 15). Isolated distension of the stomach was not observed, whereas isolated distension of either the large bowel (n = 8) or small bowel (n = 3) were less frequently observed (Fig 1). Fecalization of the terminal ileum was evident in the radiological images of three patients. No overt radiological evidence of thickened, oedematous, valvulae conniventes suggestive of volvulus was observed. Six patients had clinical and/or radiological evidence of concomitant urinary retention during an acute presentation of IPO (Supplementary Table; Fig 1). No cases of bowel perforation were recorded during the follow‐up interval of this study.

### Cause of Death in m.3243A>G Patients Presenting With IPO

Eight patients died during the study inclusion dates (patients 23–30). Four patients died of aspiration pneumonia in association with IPO (subjects 23, 24, 26, and 28), 2 died of cardiorespiratory failure (subjects 28 and 29), and 1 died of urinary tract sepsis and severe encephalopathy (subject 30). One death was attributed to spontaneous intracerebral hemorrhage, which was deemed unrelated to the underlying mitochondrial disease (subject 25). Overall median survival after definitive diagnosis of mitochondrial disease in these 8 patients was 7.0 years (range, 0.5–13.8); median survival after definitive diagnosis of an acute episode of IPO was 3.3 years (range, 0–26.3).

### Identifying Associated Factors for Development of IPO in Patients With m.3243A>G Mutation

**Figure 2:**
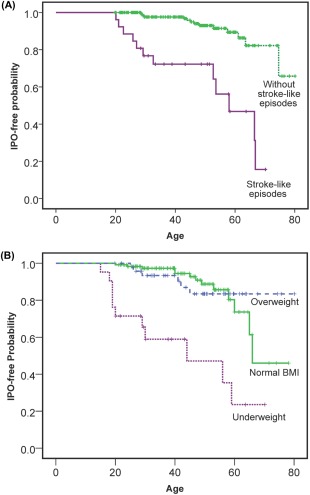
(A) Intestinal pseudo‐obstruction (IPO)‐free probability for individuals with (solid line) and without (dotted line) stroke‐like episodes. Censored data (ie, patients who have not developed IPO) at their most recent clinical assessment) are shown as crosses. The age of developing IPO is significantly lower in those with stroke‐like episodes (*p* = 0.01). (B) IPO‐free probability in three body mass index (BMI) categories: underweight in those BMI <20 (dotted line); normal weight in those BMI range 20 to 25 (solid line); and overweight in those BMI >25 (dashed line). The age of developing IPO is significantly lower in those that are underweight (*p* < 0.01). [Color figure can be viewed in the online issue, which is available at www.annalsofneurology.org.]

Median time to the development of the first clinically recognized episode of IPO was 40 years (range, 9–66). The strongest predictor for development of IPO was stroke‐like episodes (*p* = 0.01) followed by cardiomyopathy (*p* = 0.03), BMI (*p* = 0.04), and urine mtDNA heteroplasmy per 10% change (*p* < 0.01; n = 183; Table). Sex (*p* = 0.569) and presence of diabetes mellitus (*p* = 0.846) did not predict development of IPO. Survival function using stroke‐like episodes and categories of BMI are shown in Figure 2.

**Table 1:** Predictors of the Development of Intestinal Pseudo‐Obstruction Using Cox Regression Analysis (n=183)

**Predictors**	**HR**	**95% CI for HR**		***p***
		**Lower**	**Upper**	
Cardiomyopathy	2.36	1.07	5.21	0.03
Stroke‐like episodes	2.93	1.28	6.72	0.01
Body mass index	0.89	0.80	0.99	0.04
Urine heteroplasmy per 10% change	1.40	1.11	1.76	<0.01

Only patients aged 18 years or older were included for the analysis.

HR = hazard ratio; CI = confidence interval.

## Discussion

We believe that these cases of IPO highlight an important and under‐recognized clinical entity in those who carry the m.3243A>G mutation. These patients were most frequently under the care of a neurologist with known m.3243A>G‐related mitochondrial disease. They subsequently presented with gastrointestinal issues that were mistakenly thought to be unrelated to their primary mitochondrial disorder. And although our findings highlight that this can occur throughout the entire spectrum of m.3243A>G**‐**related disease, it is most commonly observed in those manifesting with high disease burden and multiorgan involvement. Only in very rare instances have such severe gastrointestinal dysmotility been the first manifestation of m.3243A>G‐related mitochondrial disease or occurred in apparently oligosymptomatic patients. The presence of preceding stroke‐like episodes, cardiomyopathy, low BMI (likely reflecting poor nutritional status), or high mtDNA heteroplasmy in patients with the m.3243A>G mutation appear cogent to the development of severe IPO, irrespective of diabetes status and sex.^21^ Moreover, retrospective review of clinical symptoms in all cases would suggest a protracted history consistent with significant, long‐standing gastrointestinal dysmotility that was often underappreciated, before the patient's acute presentation.

Although MNGIE is well recognized to cause severe gastrointestinal involvement,^14^, ^15^ our findings suggest that severe gastrointestinal involvement is much more common overall in m.3243A>G‐related mitochondrial disease. Thirteen percent of our patient cohort presented with severe gastrointestinal dysmotility symptoms with almost one third concomitantly presenting during an acute stroke‐like episode. Mid‐point 2014 census population data for the North East of England, as specified by the Office for National Statistics, UK (2,618,710),^22^ were used to estimate the prevalence rate of IPO in m.3243A>G‐related mitochondrial disease, as described previously.^2^ The minimum prevalence rate of IPO in clinically manifesting m.3243A>G carriers was estimated as 1 in 200,000 or 0.53 per 100,000 (95% confidence interval [CI]: 0.3–0.9 per 100,000), which is comparable to the previously published point prevalence of all‐cause chronic IPO (0.9 per 100,000).^23^, ^24^ Furthermore, review of our patient cohort and the literature would suggest that this late gastrointestinal complication may occur in other primary mtDNA^25^, ^26^, ^27^, ^28^, ^29^ and nuclear mutations (up to 3% in *POLG*‐related mitochondrial disease^30^, but is extremely rare. Thus, given that IPO appears relatively common in this cohort of patients (and with the m.3243A>G mtDNA mutation responsible for almost one third of all forms of adult mitochondrial disease)^2^, it is imperative for neurologists to be aware of this condition and its cardinal findings.

Dilatation of both upper and lower gastrointestinal tract was the most common radiological finding in these patients. Interestingly, 6 patients had clinical and/or radiological evidence of concomitant urinary retention during their acute admission with IPO. All patients represented subsequently with variability in site and severity of gastrointestinal dysmotility symptoms. But perhaps most striking were those 6 patients who underwent surgery. Whereas 1 patient died in the perioperative period, the remaining patients continued to manifest with symptoms of severe gastrointestinal dysmotility involving other parts of the gastrointestinal tract, including stoma and end‐stage intestinal failure. These findings concur with reported high morbidity and mortality identified in patients with *all‐cause* IPO that have undergone intestinal resection.^31^ Moreover, of the 8 patients who died during the time course of the study, aspiration pneumonia as a complication of severe gastrointestinal dysmotility may be attributed to cause of death in up to half of these patients. This is contrary to previous findings, suggesting that IPO was not a major cause of death in patients with mitochondrial disease.^27^

However, we do appreciate that discerning acute IPO from true mechanical obstruction presents many diagnostic challenges. In patients that are known to harbor the m.3243A>G mutation, we would suggest a careful review of the patient's medical history to assess for chronic symptoms of gastrointestinal dysmotility and risk factors, as outlined above. Furthermore, it is important to recognize the disparity between the patient's relative stable clinical status and the disproportionately severe abdominal distension and radiological findings.^19^, ^21^ In addition, we would advise caution in the interpretation of blood lactate levels to identify ensuing severe sepsis^32^ and tissue ischemia^33^ in hemodynamically stable patients given that mitochondrial disorders are a well‐recognized cause of lactic acidemia, with up to 70% of this patient cohort having evidence of long‐standing, elevated serum lactate. Appreciation of these caveats may help to avoid misdiagnosis, unnecessary surgical intervention, and circumvent the additional catabolic stress exerted by general anesthesia in such patients with impaired physiological reserve.

The proposed pathological mechanisms underlying the development of IPO remain diverse. In m.3243A>G‐related mitochondrial disease, it is thought to be attributable, in part, to respiratory chain deficiency of the smooth muscle defined by the presence of severe cytochrome *c* oxidase (COX) deficiency throughout the gastrointestinal tract.^34^ Other pathological findings that support the role of visceral myopathy in m.3243A>G‐related IPO include morphologically abnormal mitochondria,^10^, ^35^ moderate‐to‐high mtDNA mutation heteroplasmy level detected in the gastrointestinal tract,^36^, ^37^ and extensive microvacuolation and necrosis in the intestinal muscle layers with intact ganglion cells.^35^ We speculate there may be an additional centrally mediated mechanism given that 8 of our patients presented acutely with what appears to be a “neurogastrointestinal crisis” manifesting as concomitant severe IPO and an acute stroke‐like episode, although we advise caution in this interpretation given the small numbers involved.

Although IPO can occur in both m.3243A>G‐related mitochondrial disease and MNGIE, there are a number of useful features to help discern between the two genotypes: (1) clinical manifestations of central nervous system involvement is common in m.3243A>G‐related mitochondrial disease and rare in MNGIE; (2) brain MRI changes in m.3243A>G‐related mitochondrial disease and stroke may be “relapsing remitting” in nature whereas the leukodystrophic changes in MNGIE are often permanent^38^; (3) symptomatic, demyelinating neuropathy is a frequent finding in MNGIE whereas patients with m.3243A>G‐related mitochondrial disease may have a predominantly axonal neuropathy^39^; and (4) m.3243A>G‐related disease exhibits maternal inheritance whereas MNGIE is caused by recessive mutations in the *TYMP* gene.

Based on recently published reviews on clinical management of IPO,^21^, ^40^ the data presented here, and our Centre's clinical experience, we propose expert opinion guidelines that outline the assessment and management of patients presenting with acute IPO (available on http://www.newcastle-mitochondria.com/service/patient-care-guidelines/).

In conclusion, we would advise that all patients harboring the m.3243A>G mutation should have a brief gastrointestinal assessment at each visit to the neurology clinic. Reports of significant gastrointestinal dysmotility will require a more‐comprehensive assessment, including advice from a gastroenterologist and education and derivation of an individualized treatment plan. This will facilitate early detection and timely implementation of optimal treatment strategies to mitigate the risks of developing severe IPO. We would suggest that only by neurologists recognizing this condition in their patients with genetically determined m.3243A>G‐related mitochondrial disease will there be a significant reduction in misdiagnosis, earlier instigation of appropriate medical treatment, and avoidance of unnecessary surgical intervention and its associated poor outcome.

## Author Contributions

G.S.G., Y.N., R.M.F., and D.M.T. contributed to conception and design of the study. G.S.G., Y.N., C.F., C.E.H., J.P.G., Y.Y., A.M.S., P.H., C.L.A., M.R., M.M., A.B., R.W.T., R.M.F., and D.M.T. contributed to acquisition and analysis of data. G.S.G., Y.N., J.P.G., M.M., M.R., R.M.F., A.B., P.H., Y.Y., and D.M.T. contributed to drafting the text or preparing the figures.

## Potential Conflicts of Interest

Nothing to report.

## Supporting information

Additional supporting information can be found in the online version of this article

Supporting Information Table 1.
