# Supplementary material for: Pseudo‐obstruction, stroke, and mitochondrial dysfunction: A lethal combination
Source: Ann Neurol. 2016 Sep 19;80(5):686–92. doi: 10.1002/ana.24736 (PMC5215534; doi:10.1002/ana.24736)
Supplement: Supplementary file 1 — Supporting Information Table 1. [file ANA-80-686-s001.docx]

| Patient | IPO/ current age | BMI | Clinical feature | NMDAS | Urine mutation load (%) | Serum Lactate  (mmol/L) |
| --- | --- | --- | --- | --- | --- | --- |
| 1 (M) | 49/52 | 18.7 | MELAS syndrome, DM, deafness, LVH | 71 | 69 | 2·0-4·0 |
| 2 (F)^d^ | 66/74 | 21.9 | MIDD, myopathy, ataxia | 46 | 47 | 1·6-2·7 |
| 3 (F) | 28/29 | 18.5 | Myopathy, LVH, mild deafness | 25 | 86 | 1·0-3·9 |
| 4 (M)^a^ | 47/52 | 22.2 | MELAS syndrome, DM | 82 | 83 | 3·3-4·2 |
| 5 (F)^c^ | 29/29 | 10.6 | Exercise intolerance, myopathy | 43 | 67 | n.d. |
| 6 (F)^c,d^ | 29/29 | 27.8 | Migraine, fatigue | 33 | 52 | n.d. |
| 7 (F) | 53/57 | 24.1 | MIDD, ataxia, myopathy | 21 | 72 | 1·2-1·8 |
| 8 (M)^a^ | 19/27 | 17.1 | MELAS syndrome, LVH, deafness | 35 | 94 | 1·9-6·3 |
| 9 (F)^a^ | 19/21 | 14.3 | MELAS, failure to thrive, deafness | 45 | 82 | n.d. |
| 10 (M) | 53/53 | 27.7 | MELAS syndrome | 34 | 85 | 1·7 |
| 11 (F)^a^ | 18/26 | 17.8 | MELAS syndrome | 72 | 83 | 10·7 |
| 12 (M) | 41/44 | 24.5 | MIDD, migraine, mild LVH | 28 | 80 | 1·3-1·9 |
| 13 (F) | 44/46 | 16.5 | MIDD, seizure | 61 | 78 | 1·8-3·5 |
| 14 (F) | 40/43 | 18.6 | MIDD | 27 | 45 | 1·4-2·0 |
| 15 (F) | 25/28 | 26.7 | Deafness, macular pigmentary changes | 33 | 83 | 4·7 |
| 16 (M) | 46/48 | 25.1 | MIDD, ataxia, dilated cardiomyopathy | 68 | 88 | 3·3 |
| 17 (F)^a^ | 19/32 | 26.2 | MELAS syndrome, deafness | 38 | 91 | 1·6-1·9 |
| 18 (M)^a,d^ | 24/29 | 20.3 | MELAS syndrome, deafness, DM | 45 | 93 | 3·1-6·8 |
| 19 (M) | 9/10 | n.d. | MELAS syndrome, severe myopathy | n.d. | 99 | n.d. |
| 20 (F) | 58/63 | 18.3 | Deafness | 16 | 67 | 3·2-4·5 |
| 21 (F)^c^ | 54/54 | 19.8 | MIDD, maculopathy | 33 | n.d. | 2·5 |
| 22 (M) | 43/48 | n.d. | MIDD, LVH | 41 | 78 | n.d. |
| 23 (M)^b^ | 60/60 | 19.0 | MIDD | 52 | 35 | 3·4 |
| 24 (F)^b^ | 49/49 | n.d. | Gestational diabetes, migraine, ataxia | 34 | n.d. | n.d. |
| 25 (M)^b^ | 40/45 | 22.3 | MIDD, ESRF, renal and pancreatic transplant | 37 | 56 | 0·7-2·7 |
| 26 (M)^a,b,c,d^ | 20/20 | 16.5 | MELAS syndrome | 68 | 96 | 2·5-6·6 |
| 27 (F)^b,c^ | 56/66 | 16.6 | MELAS syndrome, deafness, DM | 60 | 55 | 2·2-3·3 |
| 28 (F)^b,d^ | 65/65 | 17.9 | MELAS syndrome, DM | 62 | 60 | 3·0 |
| 29 (M)^a,b,d^ | 20/23 | 19.0 | MELAS syndrome, dystonia | 67 | 93 | 3·2-7·0 |
| 30 (F)^b,c^ | 26/53 | 25.6 | MELAS syndrome, deafness, DM | 45 | 91 | 1·8-4·0 |

**Supplemental Table 1. Summary of demographic, clinical features and molecular genetic data of patients with m·3243A>G mutation presenting with intestinal pseudo-obstruction (n= 30).** ^a^ = intestinal pseudo-obstruction concomitant with stroke; ^b^= deceased; ^c^= surgical intervention; ^d^= acute urinary retention ; CPEO= chronic progressive external ophthalmoplegia; DM= diabetes mellitus; F= female; HCM= hypertrophic cardiomyopathy; IPO= intestinal pseudo-obstruction; LVH= left ventricular hypertrophy; M= male; MELAS= mitochondrial encephalomyopathy, lactic acidosis and stroke-like episode; MIDD= maternally inherited diabetes and deafness; n.d.= not done; NMDAS= Newcastle Mitochondrial Disease Adult Scale
